# CD109 Overexpression in Pancreatic Cancer Identified by Cell-Surface Glycoprotein Capture

**DOI:** 10.4172/jpb.S10-003

**Published:** 2014-11-18

**Authors:** Randy S Haun, Chun-Yang Fan, Samuel G Mackintosh, Hong Zhao, Alan J Tackett

**Affiliations:** 1 Central Arkansas Veterans Healthcare System, University of Arkansas for Medical Sciences; Little Rock, Arkansas, USA; 2 Departments of Pharmaceutical Sciences, University of Arkansas for Medical Sciences; Little Rock, Arkansas, USA; 3 Biochemistry and Molecular Biology, University of Arkansas for Medical Sciences; Little Rock, Arkansas, USA; 4 Department of Experimental Center of Functional Subjects, College of Basic Medicine, China Medical University, Shenyang, China

**Keywords:** Pancreatic cancer, Glycoproteins, Proteomic profiling

## Abstract

**Background:**

The development of novel targeted cancer therapies and/or diagnostic tools is dependent upon an understanding of the differential expression of molecular targets between normal tissues and tumors. Many of these potential targets are cell-surface receptors; however, our knowledge of the cell-surface proteins upregulated in pancreatic tumors is limited, thus impeding the development of targeted therapies for pancreatic cancer. To develop new diagnostic and therapeutic tools to specifically target pancreatic tumors, we sought to identify cell-surface proteins that may serve as potential tumor-specfic targets.

**Methods:**

Membrane glycoproteins on the pancreatic cancer cell lines BxPC-3 were labeled with the bifunctional linker biocytin hydrazide. Following proteolytic digestion, biotinylated glycopeptides were captured with streptavidin-coupled beads then released by PNGaseF-mediated endoglycosidase cleavage and identified by liquid chromatography-tandem mass spectrometry (MS). A protein identified by the cell-surface glycoprotein capture procedure, CD109, was evaluated by western analysis of lysates of pancreatic cancer cell lines and by immunohistochemistry in sections of pancreatic ductal adenocarcinoma and non- neoplastic pancreatic tissues.

**Results:**

MS/MS analysis of glycopeptides captured from BxPC-3 cells revealed 18 proteins predicted or known to be associated with the plasma membrane, including CD109, which has not been reported in pancreatic cancer. Western analysis of CD109 in lysates prepared from pancreatic cancer cell lines revealed it was expressed in 6 of 8 cell lines, with a high level of expression in BxPC-3, MIAPaCa-2, and Panc-1 cells. Immunohistochemical analyses of human pancreatic tissues indicate CD109 is significantly overexpressed in pancreatic tumors compared to normal pancreas.

**Conclusions:**

The selective capture of glycopeptides from the surface of pancreatic cancer cell lines can reveal novel cell-surface glycoproteins expressed in pancreatic ductal adenocarcinomas.

## Introduction

Pancreatic cancer is the fourth most common cause of cancer-related deaths in the United States [1], and is projected to be the second leading cause of cancer-related death by 2030 [2]. For over a decade, gemcitabine has been the standard of care for chemotherapy-based treatment of patients with locally advanced and metastatic pancreatic cancer, however, most studies have demonstrated low response rates and little impact on patient survival [3]. Based on the poor performance of current therapeutic modalities for pancreatic cancer, it is evident that new approaches for the treatment of this deadly neoplasm would have a major impact.

Targeted therapies are now a component of treatment for many types of cancer, including breast cancer and lymphoma. Targeted therapies may be used to 1) block the proliferation of cancer cells by interfering with specific molecules required for tumor development and growth, 2) enhance antibody-dependent cellular and complement-dependent cytotoxicity, or 3) facilitate delivery of novel nanoparticle conjugates specifically to tumor cells. Some of these targeting molecules may be present in normal tissues, but they are often mutated or overexpressed in tumors. Currently, our knowledge of the cell-surface proteins upregulated in pancreatic tumors is limited; thus impeding the development of similar targeted therapies for pancreatic cancer.

Since MS-based proteomics permit sensitive identification and quantification of large numbers of peptides or proteins, novel approaches have been developed to identify the cell- surface proteome by quantitative MS, including lectin-based methods, cell surface shaving, two-phase separation, and antibody-mediated membrane enrichment [4]. Recently, a novel method has been described for the selective isolation of N-linked glycoproteins for the analysis of the cell-surface glycoproteome, termed cell-surface capture (CSC) [4-6]. Since a compendium of such molecular targets is vital for the development of novel targeted therapies, in this study we have used the cell-surface capture procedure to specifically identify glycoproteins residing on the cell surface of a pancreatic cancer cell line, BxPC-3, and validated the identification of a cell-surface protein, CD109, in human pancreatic ductal adenocarcinoma (PDAC) tissues by immunohistochemistry (IHC).

## Materials and Methods

### Cell culture

Pancreatic cancer cell lines AsPC-1, BxPC-3, Capan-1, CFPAC-1, MIAPaCa-2, and Panc-1 were obtained from the American Type Culture Collection (ATCC, Manassas, VA). A818-4 cells were kindly provided by Professor Holger Kalthoff (Institute for Experimental Cancer Research, UKSH-Campus Kiel, Kiel, Germany) and Suit-2 cells [7] were obtained from Dr. Michael Hollingsworth (Eppley Institute, University of Nebraska Medical Center, Omaha, NE). All cells were maintained in Dulbecco's Modified Eagle's Medium (Mediatech, Manassas, VA) supplemented with 10% fetal bovine serum (Atlanta Biologicals, Norcross, GA) at 37°C in a 5% CO_2_/air environment.

### Cell-surface glycoprotein capture

For cell-surface glycoprotein capture, BxPC-3 cells were cultured in twenty 10-cm dishes to yield ∼10^8^ cells. The cells were harvested by scraping in phosphate-buffered saline (PBS), collected by centrifugation, and washed twice with labeling buffer (PBS, pH 6.5, 0.1% fetal bovine serum). To oxidize the carbohydrate moieties on the cell-surface proteins, the cells were suspended in 1.6 mM sodium *meta*-periodate (Fisher Scientific, Palantine, IL) in PBS, pH 6.5, and incubated in the dark for 15 min at 4°C. The cells were collected by centrifugation and washed twice with labeling buffer to remove residual sodium *meta*-periodate and dead cells.

The cell-surface proteins were then labeled by suspending the cells in labeling buffer containing 5 mM of the bi-functional linker biocytin hydrazide (Biotium, Hayward, CA) and incubating for 1 h at 4°C on a rotator. The labeled cells were collected by centrifugation and washed twice with labeling buffer to remove unreacted biocytin hydrazide and dead cells. The cells were then lysed by suspending the cell pellet in ice-cold hypotonic buffer (10 mM Tris-HCl, pH 7.5, 0.5 mM MgCl_2_) and disrupting the cells with a glass Dounce homogenizer. After adding an equal volume of membrane prep buffer (280 mM sucrose, 50 mM MES, pH 6, 450 mM NaCl, 10 mM MgCl_2_) to the lysates, nuclei and intact cells were removed by centrifugation at 2,500 g at 4°C for 10 min.

The homogenization procedure was repeated with the pellet and the supernatants containing the membrane fraction were combined and centrifuged at 150,000 at 4°C for 1 h. The resulting pellet was incubated with 25 mM Na_2_CO_3_, pH 11, for 30 min on ice, and then the ultracentrifugation was repeated. The labeled membrane preparation was suspended in 50 mM ammonium bicarbonate containing 0.1% of the surfactant RapiGest SF (Waters, Milford, MA) with the aid of a sonicator. The protein concentration was determined using a bicinchoninic acid (BCA) assay (Sigma-Aldrich, St. Louis, MO). Proteins were reduced with 5 mM Tris(2-carboxyethyl)phosphine hydrochloride (Sigma-Aldrich, St. Louis, MO) and alkylated with 10 mM iodoacetamide (Sigma-Aldrich, St. Louis, MO) for 30 min in the dark at 25°C. The proteins were digested for 4 h with Lys-C (1:100) (EMD Biosciences, La Jolla, CA) and subsequently with Mass Spectrometry Grade Trypsin Gold (1:20) (Promega, Madison, WI) overnight. The peptide mixture was boiled for 10 min to inactivate the proteases and protease inhibitors (Roche Complete protease inhibitor cocktail, Indianapolis, IN) were added. The biotinylated glycopeptides were captured by incubating with UltraLink Streptavidin Plus beads (Pierce, Rockford, IL), equilibrated with 50 mM ammonium bicarbonate, for 1 h on a rotator. The glycopeptides captured on the streptavidin beads were washed successively with 0.5% Triton-100 in 50 mM ammonium bicarbonate, 50 mM ammonium bicarbonate, 5 M NaCl, 100 mM sodium carbonate, pH 11, and 100 mM ammonium bicarbonate. The washed beads were incubated in 100 mM ammonium bicarbonate containing 22,500 units of PNGaseF (New England Biolabs, Beverly, MA) overnight at 37°C on a rotator to release the N-linked glycopeptides. The supernatant was collected and the beads were washed once with 50 mM ammonium bicarbonate. The combined supernatants were concentrated and desalted using UltraMicroSpin columns containing C18 resin (Nest Group, Southborough, MA). The resin was washed with 50 mM ammonium bicarbonate/0.1% TFA and the retained peptides were eluted with 70% acetonitrile/0.1% TFA.

### LC-MS/MS

The eluate from the C18 column was dried in a SpeedVac and resolubilized in 0.1% formic acid (Pierce, Rockford, IL). Tryptic peptides were separated by reverse phase Jupiter Proteo resin (Phenomenex, Torrance, CA) on a 100×0.075 mm column using a nano2D HPLC system (Eksigent, Framingham, MA). Peptides were eluted using a 45 min gradient from 97:3 to 35:65 buffer A:B ratio [buffer A=0.1% formic acid, 0.5% acetonitrile; buffer B=0.1% formic acid, 75% acetonitrile]. Eluted peptides were ionized by electrospray (2.0 kV) followed by MS/MS analysis using collision induced dissociation on an LTQ XL mass spectrometer (Thermo Scientific, Waltham, MA). MS data were acquired over a range of 375 to 1500 m/z. MS/MS data were then acquired for the top 7 peaks from each MS scan using a normalized collision energy of 35.0. Proteins were identified from MS/MS spectra by database searching using the Mascot Server 2.4 search engine (Matrix Science, Boston, MA, on-site license) with a peptide mass tolerance of 2.0 Da, fragment mass tolerance of ± 0.5 Da, a maximum of 2 missed tryptic cleavages, and fixed carbamidomethylation of cysteine modification and variable deamidation, acetylation, and oxidation modifications. The Mascot results were uploaded into Scaffold 4.3.4 (Proteome Software, Portland, OR) for viewing the proteins and peptide information. A peptide threshold of 20% with protein threshold of 95% and a minimum of one peptide were used as the cutoff values, and spectral counts were exported into an Excel spreadsheet for analysis.

### Western blot

Confluent monolayers of pancreatic cancer cell lines were harvested by scraping, washed with PBS, and collected by centrifugation. Cell pellets were suspended in RIPA buffer (1 mM EDTA, 1% NP-40, 0.5% deoxycholate, 0.1% SDS in PBS) containing Complete protease inhibitor cocktail (Roche, Indianapolis, IN). The cell lysates were sonicated on ice and centrifuged at 13,000 rpm to remove any cell debris and the protein concentration of each lysate was determined by BCA assay. Equal amounts of each sample (40 μg of total protein) were separated by gel electrophoresis using 4-12% gradient Bis-Tris NuPage gels (Life Technologies, Carlsbad, CA) and transferred to a PVDF membrane. The membrane was blocked with a 5% non-fat milk solution in PBS, pH 7.4, containing 0.1% Tween-20 (PBST) then incubated overnight at 4°C with anti-human CD109 antibody (AF4385, R&D Systems, Minneapolis, MN) diluted 1:2,000 with 2% non-fat milk/PBST. After washing with PBST, proteins were visualized by chemiluminescence using ECL plus reagent (GE Healthcare, Piscataway, NJ) and a ChemiDoc XRS image documentation system and Quantity One analysis software (Bio-Rad, Hercules, CA).

### Immunohistochemistry

Formalin-fixed, paraffin-embedded tissue blocks from 11 non-malignant pancreas and 18 PDAC tissues were prepared for immunohistochemical analysis. Representative hematoxylin and eosin-stained sections from each tissue were evaluated by microscopic analysis. Sections (4 μm) were deparaffinized and rehydrated in xylene followed by graded ethanol. Antigen retrieval was performed in a pressure cooker using 10 mM citrate, pH 6.0, for 20 minutes. Endogenous peroxidase activity was quenched by hydrogen peroxide treatment followed by serum-free protein block (DakoCytomation, Carpinteria, CA). Sections were incubated with a sheep anti-CD109 antibody (AF4385, R&D Systems, Minneapolis, MN), diluted 1:800 in antibody diluent (DakoCytomation, Carpinteria, CA), overnight at 4°C Immunoreactive staining was detected using a DAKO LSAB+ peroxidase system followed by hematoxylin counterstain. The staining intensity for the CD109 protein and the percentage of positive cells was scored by a pathologist (C-YF), and a composite score was calculated as follows. The staining intensity for the CD109 protein was assigned a score from 0 to 3 based on staining, with 0 indicating no staining; 1+, weakly positive; 2+, moderately positive; and 3+, strongly positive. The percentage of positive cells was scored as: 0, no positive cells; 1+, 1-25% positive cells; 2+, 26-50% positive cells; 3+, 51-75% positive cells; 4+, >75% positive cells. The composite score was calculated as the product of the staining-intensity score and the positive-percentage score, and thus ranged from 0 to 12. Staining with composite scores < 3 was categorized as Low, staining with composite scores 3-5 was categorized as moderate, while staining ≥ 6 was categorized as high. Images were captured with an Olympus BX41 microscope equipped with a SPOT RT color CCD camera and SPOT Advanced imaging software (Ver 5.1, SPOT Imaging Solutions, Sterling Heights, MI).

### Statistical analysis

The PDAC and Normal samples were compared statistically at a *P*<0.05 significance level for the group difference in composite IHC scores via two-sided Wilcoxon test, a generalization of the Mann-Whitney U test. All statistical analysis employed Prism 6 (GraphPad software, La Jolla, CA).

## Results

### Identification of cell-surface lycoproteins

To identify cell-surface glycoproteins on pancreatic cancer cells, we employed the CSC procedure with a pancreatic cell line, BxPC-3. MS/MS analysis of peptides from the labeled cell- surface proteins revealed 84 potential cell-surface proteins identified with >95% confidence. Table 1 summarizes the 25 proteins with at least two spectral counts that were identified from the BxPC-3 cells. Eighteen of the 25 proteins are predicted or known to be associated with the plasma membrane and organized as a single-pass, multi-pass, or GPI-linked membrane protein. Each of these 18 proteins is also predicted to be N-linked glycosylated, which is consistent with post-translational glycosylation during their transit through the Golgi apparatus toward their residence on the cell surface. Furthermore, as expected for glycoproteins labeled with biocytin in the CSC procedure, most of the peptides identified by MS from these proteins contain an NX^S/T^ T motif. The remaining seven proteins identified all lack an N-linked glycosylation motif and are not predicted to be N-linked glycoproteins; and thus, likely represent contaminating proteins retained during the purification procedure.

The protein identified with the highest spectral count, integrin beta-1, has been previously reported to be overexpressed in PDAC in numerous studies at both the mRNA [8-10] and protein [11-14] levels. The protein with the second highest spectral count, CD109, is a GPI- linked glycoprotein that is typically found on the cell surface of platelets, activated T-cells, and endothelial cells [15]. It has also been reported to be expressed in squamous cell carcinomas [16] and basal-like breast carcinoma [17]. Consistent with the selective capture of glycoproteins after labeling oxidized sugars with a hydrazide-activated biocytin, five unique CD109 peptides were identified that all possess an NXS^S/T^ glycosylation sequence (Table 2). Similar to our findings, peptides corresponding to CD109 were also identified in a screen of proteins secreted from a pancreatic cancer-derived (Panc-1) and nonneoplastic pancreatic ductal cell line (HPDE) [12], but expression of CD109 in pancreatic tumors has not previously been verified.

### CD109 is expressed in multiple pancreatic cancer cell lines

**Figure 1:**
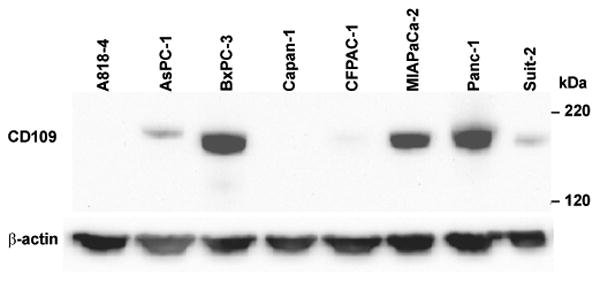
Western analysis of CD109 in pancreatic cancer cell lysates Whole-cell lysates prepared from 8 pancreatic cancer cell lines were separated by SDS-PAGE, transferred to PVDF membrane, and probed with antibodies directed against CD109 (*upper panel*) or β-actin (*lower panel*) as a loading control.

To further evaluate the expression of CD109 in pancreatic cancer, western analysis was performed on lysates prepared from 8 pancreatic cancer cell lines using a CD109 antibody (Figure 1). CD109 expression was detected in 6 of the 8 cell lines (75%), with a high level in 3 of the 8 (38%) cell lines, including BxPC-3 cells; thus, verifying the success of the MS-based glycoprotein profiling strategy. As expected, this cell-surface protein is expressed in variable levels in different cells, confirming that the use of multiple pancreatic cancer cell lines to assess cell-surface protein expression is essential for the characterization of the pancreatic cancer cell- surface glycoproteome.

### CD109 is overexpressed in pancreatic ductal adenocarcinomas

**Figure 2:**
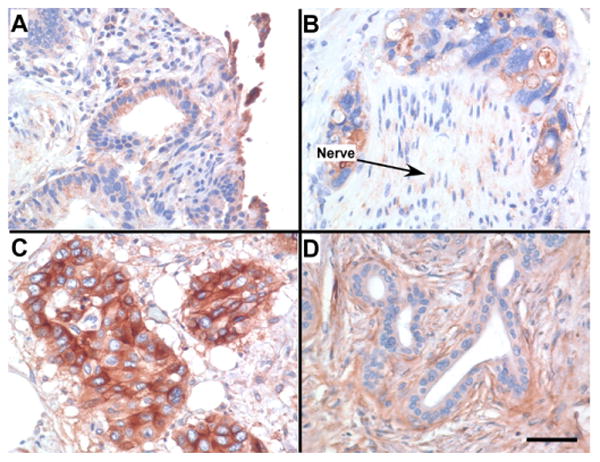
Expression of CD109 in Pancreatic Adenocarcinoma Invasive carcinoma cells were semi-quantitatively analyzed to assign the levels of CD109 protein expression as weak (**A**;+), moderate (**B**; 2+) and strong (**C**; 3+). Normal ducts from the same tumor as seen in **C** were stained negatively (**D**; -) for the CD109 protein. Perineural invasion by the tumor is seen in **B** (*arrow*). Scale bar=5 μm.

**Figure 3:**
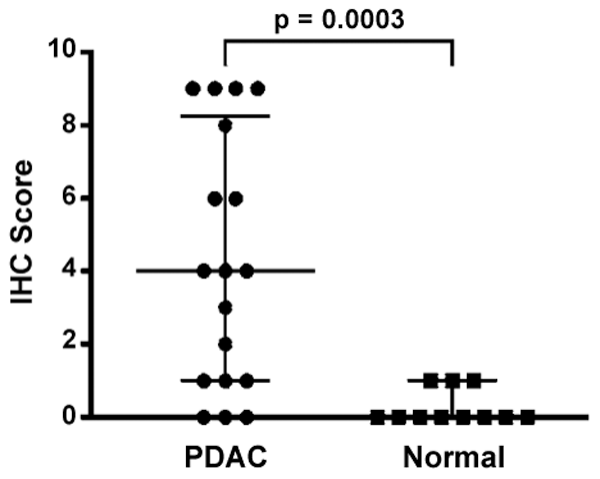
CD109 is significantly overexpressed in pancreatic ductal adenocarcinoma compared to normal pancreas tissue Intensity and distribution of staining for CD109 was evaluated in sections of pancreatic ductal adenocarcinoma (PDAC, n=18) and normal pancreas (n=11) tissue samples (see Methods). The composite IHC score determined for each sample is depicted along with the median score and interquartile range for each group (*horizontal bars*). The score difference between the groups was assessed for statistical significance at *P*<0.05 via two-sided Wilcoxon test.

We examined the cell-surface glycoproteome of BxPC-3 cells, which were established from a biopsy specimen of primary pancreatic adenocarcinoma [18], as a surrogate for human tumors. To determine whether the CD109 protein identified in the CSC procedure of BxPC-3 cells was 1) expressed in pancreatic tumors and 2) upregulated in tumors compared with normal pancreas, IHC was performed on a panel of 18 tissues sections with PDAC and 11 sections with normal pancreas tissue. As depicted in Figure 2, variable CD109 protein expression was observed in the invasive carcinoma cells, ranging from weak (panel A), moderate (panel B), to strong staining (panel C). The positive staining reaction was observed primarily along the cytoplasmic membrane and in the cytoplasm of the carcinoma cells (Figure 2A, B and C). By contrast, most normal ductal components within PDAC and in normal pancreatic tissue showed either completely negative stain for the CD109 protein (Figure 2D) or rare cases showing only focal and weak immunoreactivity for the protein. The antibody appears to be very specific for the ductal epithelia of cancer cells because it did not react with other pancreatic tissue components, such as blood vessels, stromal fibrous tissues, pancreatic acini, adipose tissue and inflammatory cells. A total of 3 of 11 (22%) of normal pancreatic tissue showed focal and weak immunostaining for CD109 (Figure 3). Moderate-to-high CD109 expression was detected in 4 of 18 (22%) and 7 of 18 (39%) PDAC specimens, respectively (Figure 3). Thus, a majority (11 of 18; 61%) of the PDAC showed significantly higher expression of CD109 compared to normal ducts in normal pancreatic tissue. The varied expression of CD109 observed in the pancreatic cancer cell lines (Figure 1) is thus very similar to the protein expression detected in human tumor specimens. Although high levels of CD109 have been reported in glioblastoma and squamous cell carcinoma cell lines [16], to our knowledge our findings represent the first description of the upregulation of CD109 in pancreatic cancer.

## Discussion

Numerous gene profiling studies, including our own [19], have been performed to identify genes differentially expressed in pancreatic tumors and cell lines [8,20-35]. Although these studies have increased our understanding of molecular pathways activated or suppressed in pancreatic adenocarcinomas, transcription analyses do not necessarily correlate with protein abundance, and thus provide only an indirect measure of alterations in protein levels [36]. Furthermore, the ability to identify cell-surface resident proteins in these studies is indirect and highly dependent upon the quality of the gene annotation data associated with the bioinformatic analyses, and may not adequately reflect the proteins displayed on the cell surface. Recently, advances in mass spectrometry (MS)-based methods have provided a direct means to profile cell-surface proteins; thus, we utilized a cell-surface capture procedure to selectively isolate and identify N-linked glycoproteins on the cell surface of a pancreatic cancer cell line which was used as a surrogate for human pancreatic ductal adenocarcinoma.

MS analysis of glycopeptides captured from the surface of BxPC-3 cells revealed the presence of glycoproteins previously associated with pancreatic cancer (e.g., integrin beta-1, intercellular adhesion molecule 1) as well as novel pancreatic cancer cell-surface proteins (e.g., CD109). The majority of the glycoproteins identified (72%) are predicted to reside on the cell surface (e.g., type I or type II membrane proteins) and have N-linked glycosylation sequences, which highlights the ability of the CSC procedure to specifically enrich for glycoproteins.

Since the expression of CD109 has not been thoroughly investigated in pancreatic cancer previously, we examined its expression in lysates of 8 pancreatic cancer cell lines by western analysis. Consistent with its MS-based identification in BxPC-3 cells following CSC, a high level of CD109 expression was observed in the BxPC-3 cell lysate. Variable levels of expression were observed in other pancreatic cancer cell lines, including a lack of expression in A818-4 and Capan-1 cells. This indicates that multiple cell lines must be used to generate a comprehensive catalog of cell-surface proteins expressed in pancreatic cancer.

Since immortalized cancer cell lines only serve as surrogates for human tumors, we examined CD109 expression in a series of human PDAC tumor and normal pancreas tissue sections by IHC. These studies indicated that CD109 is significantly overexpressed in the invasive carcinoma cells of the PDAC specimens compared to normal pancreatic tissue. Similar to the pancreatic cancer cell lines, variable CD109 protein expression was also observed in the pancreatic tumors, which is expected based on the genetic heterogeneity demonstrated among individual PDAC tumors [37]. These findings suggest that potential therapeutic targets revealed by cell-surface glycoprotein profiling may only serve a subset of patients and underscores that personalized therapeutic interventions will likely be required to target tumor-specific glycoproteins.

Overall, these results confirm that the CSC procedure was highly effective in identifying novel cell-surface proteins in pancreatic cancer and that the profiling the glycoproteome can provide valuable insights into the proteins upregulated on the surface of pancreatic tumors that may serve as targets for the development of novel diagnostic tools and/or therapies for this devastating disease.

## Figures and Tables

**Table 1:** Summary of proteins identified by cell-surface capture.

UniProt ID	Gene Symbol	Name	Location^a^	Membrane Organization^b^	N-linked^c^	NXS/T^d^	Spectral Count
P05556	ITGB1	Integrin beta-1	PM	Type I	Y	7/7	7
Q6YHK3	CD109	CD109 antigen	PM	GPI	Y	6/6	6
P05362	ICAM1	Intercellular adhesion molecule 1	PM	Type I	Y	4/5	5
P06731	CEACAM5	Carcinoembryonic antigen- related cell adhesion molecule 5	PM	GPI	Y	3/5	5
P09758	TACSTD2	Tumor-associated calcium signal transducer 2	PM	Type I	Y	3/3	3
P13726	F3	Tissue factor	PM	Type I	Y	3/3	3
P35527	KRT9	Keratin, type I cytoskeletal 9	Extracellular			0/3	3
P35613	BSG	Basigin	PM	Type I	Y	2/2	2
Q14126	DSG2	Desmoglein-2	PM	Type I	Y	2/2	2
Q08722	CD47	Leukocyte surface antigen CD47	PM	Multi-pass	Y	2/2	2
P17301	ITGA2	Integrin alpha-2	PM	Type I	Y	2/2	2
P08473	MME	Neprilysin, CD10	PM	Type II	Y	2/2	2
P04156	PRNP	Major prion protein	PM	GPI	Y	2/2	2
Q15758	SLC1A5	Neutral amino acid transporter B(0)	PM	Multi-pass	Y	2/2	2
P26006	ITGA3	Integrin alpha-3	PM	Type I	Y	2/2	2
Q9Y639	NPTN	Neuroplastin	PM	Type I	Y	2/2	2
P06756	ITGAV	Integrin alpha-V	PM	Type I	Y	2/2	2
Q9H0X4	ITFG3	Integrin alpha FG-GAP repeat containing 3	PM	Type II	Y	2/2	2
P08195	SLC3A2	4F2 cell-surface antigen heavy chain	PM	Type II	Y	1/2	2
Q9Y2I7	PIKFYVE	1-phosphatidylinositol 3-phosphate 5-kinase	Endo		N	0/2	2
Q86XA9	HEATR5A	HEAT repeat-containing protein 5A			N	0/2	2
Q8IVF2	AHNAK2	Protein AHNAK2	Nucleus		N	0/2	2
P04264	KRT1	Keratin, type II cytoskeletal 1	PM		N	0/2	2
P33778	HIST1H2BB	Histone H2B type 1-B	Nucleus		N	0/2	2
Q92529	SHC3	SHC-transforming protein 3	Cytosol		N	0/2	2

a PM–Plasma membrane, Endo–Endosomal membrane

b Type I – Type I membrane protein, Type II–Type II membrane protein, Multi-pass–Multi-pass membrane protein, GPI–Glycosylphosphatidylinositol-anchored membrane protein

c Protein predicted to have N-linked glycosylation

d Number of peptides identified with NX^S/T^ glycosylation motif/Number of peptides identified

**Table 2:** CD109 peptides identified by MS/MS.

Sequence^a^	Mascot Ion Score	Start Position	Stop Position	Modifications
(K)TASNLTVSVLEAEGVFEK(G)	52.12	65	82	
(K)TASNLTVSVLEAEGVFEK(G)	25.9	65	82	
(R)TQDEILFS**N**STR(L)	28.94	110	121	Deamidated (+1)
(R)**N**YTEYWSGSNSGNQK(M)	67.41	397	411	Deamidated (+1)
(K)INYTVPQSGTFK(I)	28.25	418	429	
(K)Q**N**ST**M**FSLTPENSWTPK(A)	70.75	512	528	Deamidated (+1) Oxidation (+16)

a Deamidated asparagine and oxidized methionine residues in **bold**, N-glycosylation motif underlined, amino acids in parentheses indicate residues preceding and following trypsin cleavage

## Abbreviations

BCA: Bicinchoninic Acid
CSC: Cell-Surface Capture
IHC: Immunohistochemistry
MS: Mass Spectrometry
PBS: Phosphate-Buffered Saline
PDAC: Pancreatic Ductal Adenocarcinoma
